# The coordinated activities of collagen VI and XII in maintenance of tissue structure, function and repair: evidence for a physical interaction

**DOI:** 10.3389/fmolb.2024.1376091

**Published:** 2024-03-28

**Authors:** Carl A. Gregory, Jocelyn Ma, Sebastian Lomeli

**Affiliations:** Department of Medical Physiology, Texas A&M School of Medicine, Bryan, TX, United States

**Keywords:** extracellular matrix, collagen, collagen VI, collagen XII, transforming growth factor inducible protein, regenerative, healing

## Abstract

Collagen VI and collagen XII are structurally complex collagens of the extracellular matrix (ECM). Like all collagens, type VI and XII both possess triple-helical components that facilitate participation in the ECM network, but collagen VI and XII are distinct from the more abundant fibrillar collagens in that they also possess arrays of structurally globular modules with the capacity to propagate signaling to attached cells. Cell attachment to collagen VI and XII is known to regulate protective, proliferative or developmental processes through a variety of mechanisms, but a growing body of genetic and biochemical evidence suggests that at least some of these phenomena may be potentiated through mechanisms that require coordinated interaction between the two collagens. For example, genetic studies in humans have identified forms of myopathic Ehlers-Danlos syndrome with overlapping phenotypes that result from mutations in either collagen VI or XII, and biochemical and cell-based studies have identified accessory molecules that could form bridging interactions between the two collagens. However, the demonstration of a direct or ternary structural interaction between collagen VI or XII has not yet been reported. This Hypothesis and Theory review article examines the evidence that supports the existence of a functional complex between type VI and XII collagen in the ECM and discusses potential biological implications.

## 1 Introduction

*Glossary of Terms* provided in Supplementary Material.

### 1.1 The extracellular matrix and the collagen family of proteins

#### 1.1.1 The extracellular matrix

The extracellular matrix (ECM) is a biomolecular network consisting primarily of proteins and glycosides that maintains structural integrity and organization of tissue. In this capacity, the ECM provides a durable 3-dimensional structure for the attachment, arrangement and orientation of cells, and it delivers biomechanical and biochemical stimuli to cells through direct attachment to ECM constituents or the via the growth factors it sequesters (Mecham, 2012; Yue, 2014). In general, ECM networks can be crudely divided into those that form fibrillar networks, sheets, and hydrogels. Approximately 300 “matrisomal” proteins have been identified in mammalian tissues, and while specific composition is tissue dependent, all possess collagen to some degree (Hynes and Naba, 2012).

#### 1.1.2 The collagen family of proteins

The collagens are a 28 member-strong family of structurally distinct proteins representing the most abundant protein subtype in mammals (Ricard-Blum, 2011). Different collagen family members have a range of relative abundancies in tissues, and all play a crucial role in the maintenance of tissue architecture. Collagen family members possess a diverse array of structural properties that facilitate formation of fibrillar structures that afford tensile strength and weight-bearing capacity (*e.g.*, cartilage, bone), woven sheets that serve as cellular attachment sites (*e.g.*, basement membranes, stem cell niche structures), and semi-permeable membranes that regulate passage of fluids and macromolecules (*e.g.*, glomerular basement membrane). Collagen molecules are modular in nature affording precise adaptation to a wide range of specific functions, but all collagens possess one common and defining feature, the triple-helix. The combination of triple-helical structural elements with globular cell and ligand-interacting motifs permit simultaneous regulation of attached cells with biomechanical and biochemical stimuli.

#### 1.1.3 The triple-helix

**FIGURE 1:**
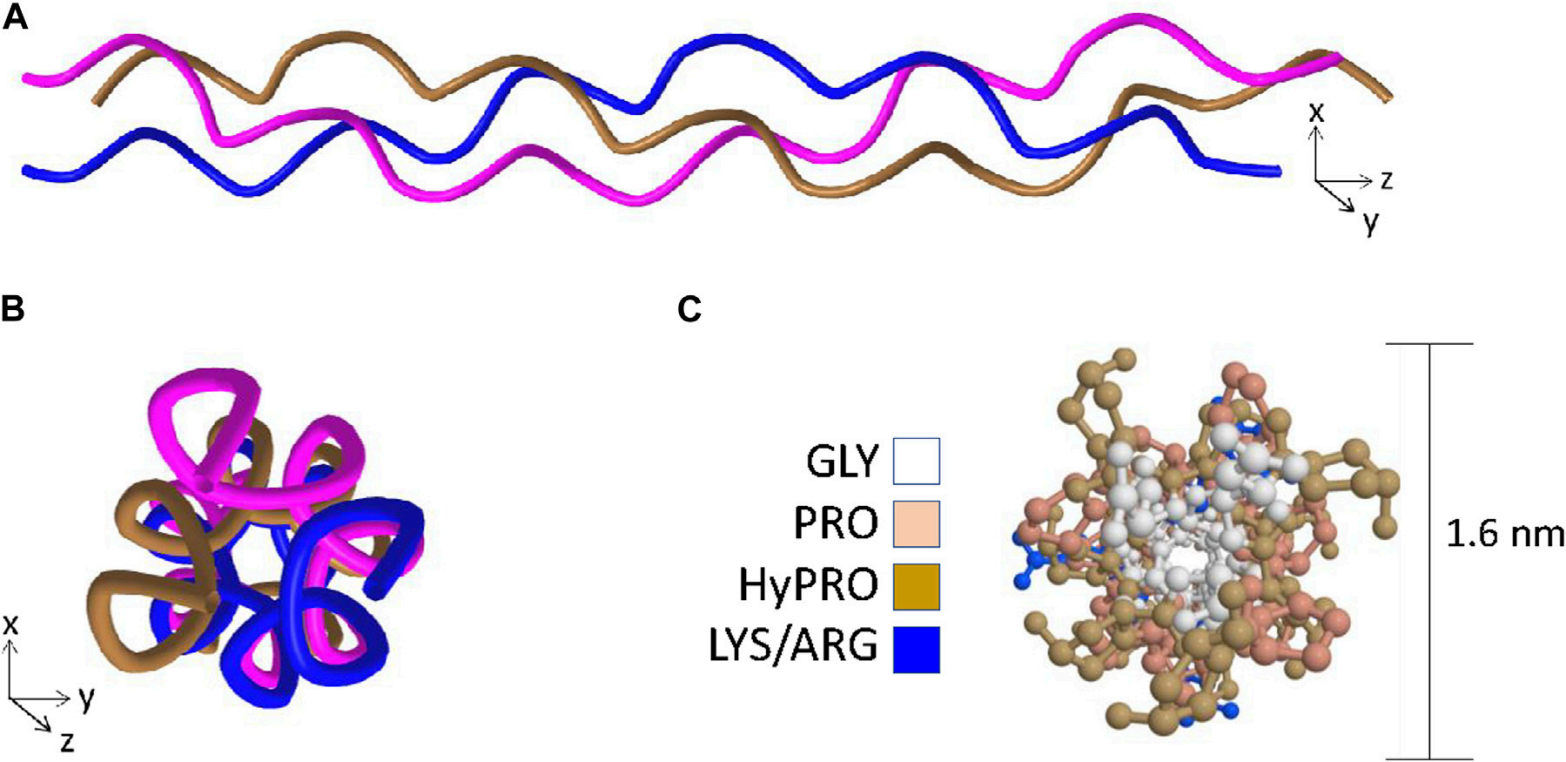
The key features of the triple-helix. **Panel (A)** Ribbon structure of the triple-helix highlighting the left-handed helix adopted by each of the individual chains and the right-handed super-helix formed when the left-handed helices wrap around one another. **Panel (B)** View in the axial orientation. **Panel (C)** Axial view with side-chains added and color coded to visualize the central positioning of the glycine residues and the outer positioning of prolines and hydroxyprolines. Structural data recovered from the Protein databank, accession 3wn8, Okoyuma *et al.* (Okuyama et al., 2014) and figure generated by MDL Chime.

Fully assembled collagen molecules are trimers of three identical or different polypeptides referred to as α-chains. A triple-helical domain is generated when each constituent α-chain forms a left-handed helix which in turn wraps around two other α-chains to form a right-handed super-helix (Figure 1A) (Ramachandran and Kartha, 1954; Bella et al., 1994; Bella et al., 1995; Okuyama, 2008). To adopt this tertiary structure, the primary structure of the triple helical domain is limited to a three residue repeat glycine-X-Y where X and Y are frequently proline and hydroxyproline residues respectively (Ramachandran and Kartha, 1954; Bella et al., 1994). The proline rings are necessary to introduce kinks in the nascent α-chains at an angle that favors formation of the left-handed helix (Bella et al., 1994; Rainey and Goh, 2002) and the glycine residues are necessary because they are the only residues compact enough to embed themselves into the center of the right handed super-helix (Figures 1B, C) (Ramachandran and Kartha, 1954; Rainey and Goh, 2002; Okuyama et al., 2014). Triple-helices are rod-shaped structures, and in the case of the abundant fibrillar collagens such as type I and II with long uninterrupted triple-helical regions, individual collagen molecules readily form supramolecular arrays that ultimately become the collagen fibrils that provide tensile strength in tissues such as cartilage, ligament, and bone (Exposito et al., 2010).

#### 1.1.4 Globular domains in collagen molecules

In contrast to the fibrillar collagens that are predominantly triple-helical in structure, many members of the collagen family possess additional non-collagenous domains that confer biological activities. These non-collagenous domains, often repeated in tandem, provide attachment sites for ECM components, sites for sequestration of growth factors, and attachment sites for cells that in some cases deliver intracellular signals (Nelson and Bissell, 2006; Ricard-Blum, 2011; Dzobo and Dandara, 2023). Fibronectin type III (F3) repeats, Kunitz domains, thrombospondin-1 (TSP-1) domains, and von Willebrand factor A (VWA) domains are the most abundant non-collagenous domains in the collagen family. F3 repeats consist of seven β-strands that form two sheets reminiscent of the immunoglobin superfamily. F3 domains readily bind proteoglycans such as heparin and possess cell recognition sites including RGD motifs capable of engaging integrin receptors (Sharma et al., 1999). Von Willebrand factor is a major component of the hemostatic pathway composed of four repeated domains designated (A-D) and it is the VWA domains that are also present in some ECM proteins. VWA domains are related to the α/β dinucleotide-binding-fold (Rosman fold) and slight sequence variations equip some VWA domains to facilitate integrin receptor-like binding of triple-helical motifs (Hohenester and Engel, 2002; Whittaker and Hynes, 2002; Becker et al., 2014). Thrombospondins are calcium binding glycoproteins with a variety of pleiotropic roles including regulation of angiogenesis, connective tissue organization and synaptogenesis (Neame et al., 1990; Bork, 1992; Carlson et al., 2005; Adams and Lawler, 2011). The function of the TSP-1 domain in collagens is unclear, but it may play a role in growth factor sequestration because TSP-1 domains in laminin have been reported to bind vascular endothelial growth factor (VEGF) and platelet derived growth factor (PDGF), significantly enhancing their efficacy in wound healing models (Ishihara et al., 2018). The TSP-1 domain also has the capacity to bind α4-integrin (Calzada et al., 2004).

### 1.2 Type VI collagen

#### 1.2.1 Collagen VI structure

**FIGURE 2:**
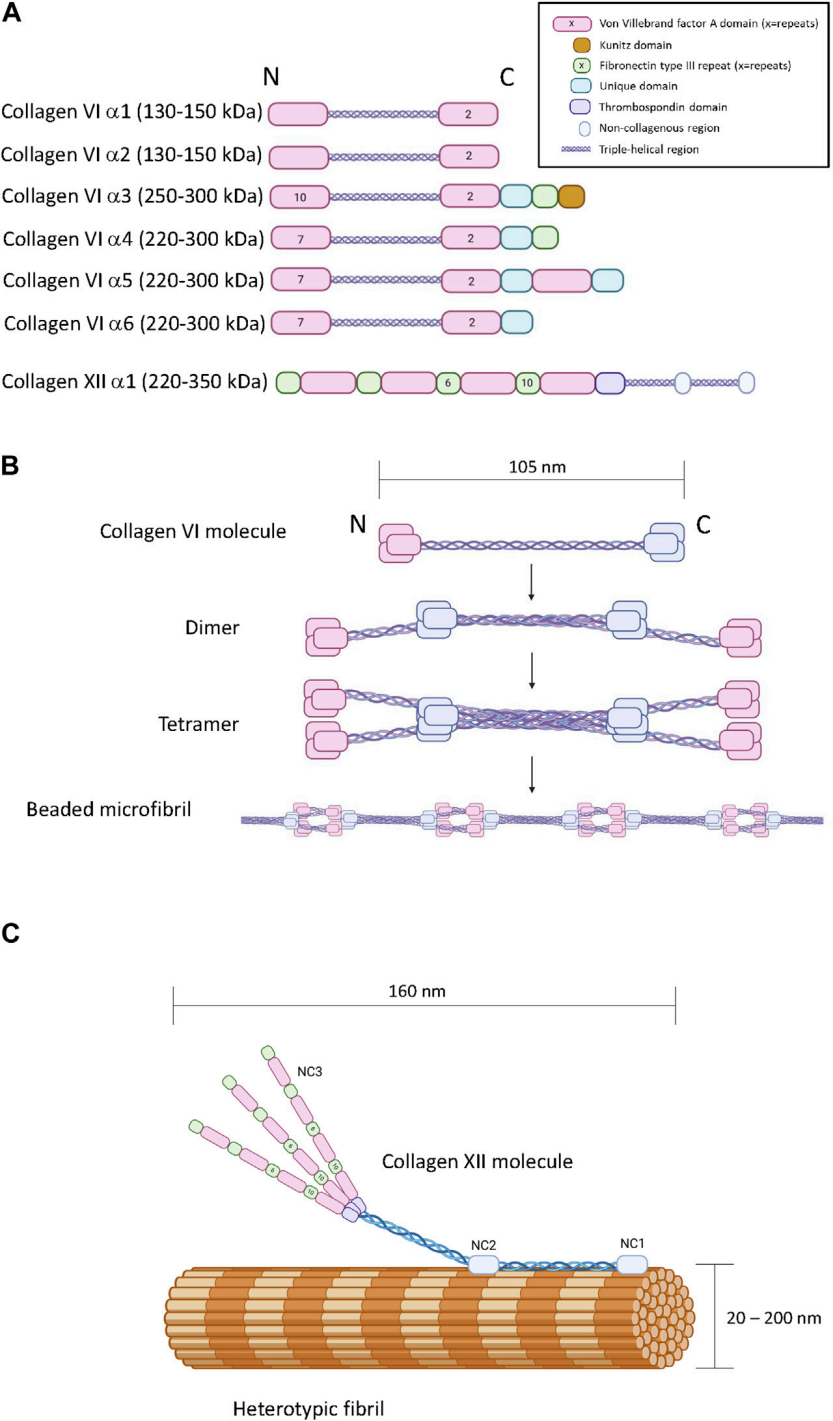
The primary, quaternary and tertiary structure of type VI and type XII collagen. **Panel (A)** Schematic diagram of the modular primary sequence of the type VI collagen α-chains and the type XII collagen α1 chain. Note that the numbers within each module refer to the number of repeats. Observed molecular masses are provided in parentheses. **Panel (B)** Formation of beaded microfilaments by supramolecular assemblies of type VI collagen dimers and tetramer. **Panel (C)** Incorporation of type XII collagen into heterotypic fibrils. Generated by Biorender.

There are six COL6A genes in the human genome (*COL6A1-6*). The predominant form of the collagen VI molecule is a heterotrimer of α1(VI), α2(VI) and α3(VI) chains (Cescon et al., 2015). In the absence of a functional α3(VI) chain, α1(VI), α2(VI) alone cannot form viable collagen VI heterotrimers indicating that the α3(VI) chain is necessary for successful assembly and secretion of the common form of collagen VI (Lamandé et al., 1998). The *COL6A4, COL6A5* and *COL6A6* genes generate α(VI) polypeptides that are similar in size and structure to the α3(VI) chain suggesting functional complementarity (Figure 2A) (Fitzgerald et al., 2008; Gara et al., 2008; Fitzgerald et al., 2013). In transfection studies, murine α4(VI) successfully assembles into heterotrimers with α1(VI) or α2(VI) chains (Fitzgerald et al., 2008) and experiments on recombinant and tissue-derived collagen VI in mice suggest that α4(VI), α5(VI) and α6(VI) can form mixed trimers with α1(VI) and α2(VI) (Maass et al., 2016). Due to a genetic rearrangement, human *COL6A4* transcripts are not translated (Fitzgerald et al., 2008), dismissing the possibility of human α1(VI), α2(VI), α4(VI) trimers. The potential for α5(VI) and α6(VI) to serve as a replacement for α3(VI) in human tissues is unclear because human α5(VI) and α6(VI) chains do not generate secreted heterotrimers with α1(VI) or α2(VI) in SAOS cells that lack the capacity to express α3(VI) (Fitzgerald et al., 2008). However, it should be noted that these experiments are limited because they do not reflect the complexity of intact tissue, and while α5(VI) and α6(VI) chains are present in a variety of human tissues (Veeman et al., 2003), the specific trimeric composition is not known.

In terms of chain structure, the type VI collagen alpha chains all possess at least three VWA domains, and the α3(VI) - α6(VI) chains also share unique domains characterized by a short cysteine-containing region of homology. The α3(VI) and α4(VI) chains also contain a F3 domain at the carboxyl terminus (Fitzgerald et al., 2013). A carboxyl-terminus Kunitz protease inhibitor domain in the α3(VI) chain is required for supramolecular assembly but subsequently cleaved (Lamande et al., 2006) (Figure 2A). Interestingly, the Kunitz domain of the α3(VI) chain probably has bioactivity in the ECM after cleavage, reportedly stimulating inflammation, insulin resistance, fibrosis and tumor progression, also referred to as endotropin (Heumuller et al., 2019).

Collagen VI heterotrimers undergo supramolecular assembly to form beaded microfibrils (Figure 2B). During this multistage process, two collagen VI heterotrimers associate together to form antiparallel dimers stabilized by disulphide bonding (Baldock et al., 2003; Cescon et al., 2015). Elements in the α2(VI) chain are specifically required for this process, including a metal-ion coordination site in the second carboxyl-terminal VWA domain and an integrin binding site at the amino-terminal end of the triple-helical domain of the other participant of the dimer (Ball et al., 2003). Collagen VI dimers then align to form tetramers which are further stabilized by disulphide bone formation (Colombatti et al., 1995). Unlike most other collagens, this tetramerization process proceeds inside the cell, followed by secretion into the extracellular space where the tetramers further self-assemble into long microfilaments characterized by bead repeat of 105 nm corresponding to the clusters of amino and carboxyl terminal domains (Knupp et al., 2006; Godwin et al., 2017). The fifth VWF module in the α3(VI) chain is essential for the assembly of tetrameric monomers into beaded microfibrils (Fitzgerald et al., 2001).

#### 1.2.2 Collagen VI expression pattern

Collagen VI expression initially occurs in developing skin, eye, spleen, heart, kidney, and skeletal muscle (Kielty et al., 1993; Magro et al., 1996; Gittenberger-de Groot et al., 2003; Tonelotto et al., 2019) with heavy expression at sites of ossification, articular chondrogenesis, and odontogenesis (Quarto et al., 1993; Salter et al., 1995; Marvulli et al., 1996; Mammoto et al., 2015). In adults, collagen VI consisting of α1, α2, and α3 chains is broadly expressed with greatest representation in connective tissues including bone, skin, tendon and cartilage (Pullig et al., 1999; Watson et al., 2001; Di Martino et al., 2023), interstitial fibroblasts of skeletal muscle (Nishimura et al., 1997; Braghetta et al., 2008; Zou et al., 2008), and in central and peripheral nervous system (Genecards, 2024), intestine (Genecards, 2024), lung (Genecards, 2024), adipose tissue (Genecards, 2024), pancreatic islets (Hughes et al., 2006), ovarian follicles, kidney glomeruli (Razzaque et al., 1999), vasculature (Hitraya et al., 1995), and cornea (Dua et al., 2023). When analyzed by immunohistochemistry, the tissue distribution of α4(VI) and α6(VI) chains is somewhat more restricted than colα3(VI) with greatest representation in intestine, ovary and testes for α4(VI), and eye, heart, lung, and skeletal muscle for α6(VI). The α5(VI) chain is more widely distributed, detectable in eye, heart, intestine, skeletal muscle, kidney, gonads, skin, and blood vessels. Notably, expression of the α4(VI), α5(VI), α6(VI) chains is marginal in bone and cartilage (Gara et al., 2011).

#### 1.2.3 Collagen VI function

The primary function of collagen VI beaded fibrils is to serve as an interface between cells and the interstitial ECM as a component of the pericellular ECM (Macri et al., 2007; Osidak et al., 2015; Zelenski et al., 2015; Bandzerewicz and Gadomska-Gajadhur, 2022). While adopting this role, it serves to transduce mechanosensory signals from the ECM to cells in load-bearing tissues such as cartilage (Zelenski et al., 2015), tendon (Sardone et al., 2016) and muscle (Urciuolo et al., 2013), provides a cellular anchor to basement membranes (Kuo et al., 1997), and drives matrix assembly during wound healing (Lettmann et al., 2014; Theocharidis et al., 2016). Collagen VI also has the capacity to regulate the activity, retention and survival of progenitor cells in a variety of tissues such as bone (Izu et al., 2016; McNeill et al., 2020), adipose (Nakajima et al., 2002) muscle (Urciuolo et al., 2013), hematopoietic (Klein et al., 1995), neural (Gregorio et al., 2018), and epithelium (Takagi et al., 2012). Collagen VI probably performs the majority of its signaling functions via direct engagement of cell surface receptors such as integrins and neuron-glial antigen 2 (NG2)/chondroitin sulphate proteoglycan 4 (CSPG4) receptor (Doane et al., 1998; Di Martino et al., 2023), but it also has the means to sequester cell-signaling ligands such as PDGF (Somasundaram and Schuppan, 1996; Ishihara et al., 2018), oncostatinM (Somasundaram et al., 2002), bone morphogenic protein 7 (BMP7) (Vukicevic et al., 1994), and matrix-metalloproteinases (Freise et al., 2009) thereby affecting cell function through regulation of the availability of these factors. Collagen VI also regulates fundamental cellular processes such as apoptosis, proliferation, and autophagy (Cescon et al., 2015; Castagnaro et al., 2021; Di Martino et al., 2023).

#### 1.2.4 Collagen VI mutations and disease

Dominant and recessive mutations in type VI collagen cause a range of muscular dystrophies with various levels of severity, most notably Bethlem myopathy at the milder end of the spectrum with Ullrich congenital muscular dystrophy representing the more severe forms (Foley et al., 1993; Jobsis et al., 1996; Camacho Vanegas et al., 2001; Baker et al., 2005; Bonnemann, 2011; Bushby et al., 2014). Collagen VI dysfunction is thought to contribute to myopathy by compromising the anchorage of myoblasts to the muscle basement membrane and by interfering with the response of myoblast progenitors (satellite cells) to tissue wear and injury. Skin and tendon is also affected by collagen VI deficiency through abnormal interactions between resident cells and collagen I containing fibrils, resulting in their aberrant synthesis and weakened tissue (Lamande and Bateman, 2018). Collagen VI mutations have also been shown to adversely affect the structure and function of mitochondria potentially via aberrant integrin signaling (Irwin et al., 2003) and the capacity to undergo natural autophagy in muscles (Pan et al., 2014; Lamande and Bateman, 2018) suggesting a metabolic phenotype in these diseases too.

In addition to the collagen VI dystrophies, a *COL6A6* missense mutation has been associated with retinitis pigmentosa (Vaclavik et al., 2022), a missense in *COL6A2* has been associated with progressive myoclonus epilepsy syndrome (Karkheiran et al., 2013), a *COL6A5* variant has been associated with chronic itch disorder (Martinelli-Boneschi et al., 2017) and several mutations have been associated with isolated recessive dystonia (Domingo et al., 2016; Lohmann et al., 2016). Furthermore, polymorphisms have been linked to atopic dermatitis (Jung et al., 2022) and corneal resistance (Van Hout et al., 2020). While knockout mouse models exhibit clear skeletal and cartilage dysfunctionality (Quarto et al., 1993; Christensen et al., 2012; Zelenski et al., 2015), collagen VI mutations in humans are associated with surprisingly few disorders that affect the skeleton directly. Nevertheless, certain collagen VI polymorphisms have been reported to predispose humans to pathological ossification of the posterior longitudinal ligament (Wang et al., 2019) and osteoarthritis (Gari et al., 2016).

### 1.3 Type XII collagen

#### 1.3.1 Collagen XII structure

Collagen XII is a member of the fibril-associated collagens with interrupted triple-helices (FACIT) family of collagens. A single *COL12A1* gene encodes a polypeptide with two collagenous domains separated by three non-collagenous domains (NC1, NC2 and NC3) (Figure 2A) (Gordon et al., 1990). The amino-terminal NC3 domain consists of 18 F3 repeats and 4 VWA repeats (Figure 2C) (Izu and Birk, 2023), but splice variation can result in the synthesis of an additional form truncated between the seventh and eighth F3 modules referred to as collagen XIIB, where the full-length variant is referred to as collagen XIIA (Chiquet et al., 2014). Collagen XII can exist as homotrimers and heterotrimers of collagen XIIA and XIIB and the relative abundance of homotrimers and heterotrimers appears to be tissue specific (Koch et al., 1995). While distinctive functions of the collagen XIIA and B forms are currently unclear, it is noteworthy that the NC3 domain of Collagen XIIA can be glycosylated whereas collagen XIIB is not, and the splice variants possess different affinities for heparin (Koch et al., 1995). There is no clear evidence that collagen XII trimers form higher order structures with themselves, but its incorporation into heterotypic fibrils consisting of type I and type II collagen has been heavily documented with biochemical and microscopic evidence indicating direct incorporation of the triple helical domains into the fibril with exposure of the NC3 domain (Figure 2C) (Keene et al., 1991; Koch et al., 1995; Chiquet et al., 2014). Interactions with non-collagenous ECM molecules such as tenascins, decorin, fibonectin, transforming growth factor induced protein (TGFIP), and cartilage oligomeric matrix protein (COMP) have also been reported (Agarwal et al., 2012; Runager et al., 2013; Izu and Birk, 2023).

#### 1.3.2 Collagen XII expression

Collagen XII is co-expressed developmentally with collagen I and II - containing fibrils in connective tissues (Watt et al., 1992; Oh et al., 1993; Walchli et al., 1994; Koch et al., 1995; Gregory et al., 2001; Chiquet et al., 2014; Izu and Birk, 2023) such as tendon/ligament, growth plate and articular cartilage (Watt et al., 1992; Gregory et al., 2001), skin, smooth muscle (Chiquet et al., 2014) and bone (Chiquet et al., 2014), and also in endothelial and epithelial basement membranes (Thierry et al., 2004) and cornea (Gordon et al., 1996). In healthy adult tissue, expression is more restricted to collagen I - containing fibrils found in tissues such as bone (Izu et al., 2011; Izu et al., 2016; Mondragón et al., 2020; Izu and Birk, 2023), tendon/ligament (Izu et al., 2021; Fung et al., 2022; Izu and Birk, 2023), cornea (Anderson et al., 2000; Espana and Birk, 2020; Sun et al., 2020), and skin (Berthod et al., 1997; Schonborn et al., 2020). Expression of collagen XII is also coincident with regenerative processes in a wide range of adult tissues including bone (Zeitouni et al., 2012; McNeill et al., 2020), tendon/ligament (Thomopoulos et al., 2002; Tsuzuki et al., 2016), cardiac muscle (Marro et al., 2016), skin (Brant et al., 2015; Schonborn et al., 2020), cornea (El-Shabrawi et al., 1998; Donovan et al., 2023), and even spinal cord (Wehner et al., 2017). In the newt, collagen XII is broadly expressed in the blastema during epimorphic regeneration of appendages (Wei and Tassava, 1996). Collectively, these expression patterns suggest that collagen XII may play multiple roles in *de novo* ECM assembly associated with development and healing.

#### 1.3.3 Collagen XII function

While the biochemical factors controlling collagen XII expression have not been definitively elucidated, it is known to be upregulated in response to mechanical stimuli in fibroblasts (Trachslin et al., 1999), osteoblasts (Arai et al., 2008), ligament (Karimbux and Nishimura, 1995), and muscle (Fluck et al., 2000). Not surprisingly, an enhancer responsive to static tensile strain has been identified in the chick collagen XII promoter (Chiquet et al., 1998) and another enhancer responsive to cyclic strain has been identified in the murine collagen XII promoter (Arai et al., 2008). It is noteworthy that collagen XII expression is tightly regulated in tissues that typically remodel in response to biomechanical stimuli, providing additional credence to the notion that collagen XII plays a key role in *de novo* ECM assembly. Accordingly, studies of collagen I-rich tissues of wild-type and collagen XII deficient mice confirm that collagen XII participates in fibrillogenesis by regulating spacing, crosslinking, and assembly of heterotypic fibrils (Young et al., 2002; Zvackova et al., 2017; Schonborn et al., 2020; Fung et al., 2022). Collectively, the data indicate that collagen XII increases the stiffness and durability of the ECM through participation in interfibrillar cross-linking (Nishiyama et al., 1994; Turko et al., 2017; Nair et al., 2022), and has the capacity to transduce mechanical signals back to attached cells (Chiquet et al., 1996). Collagen XII also regulates the biomechanical characteristics and chemical composition of the ECM by serving as a depot for transforming growth factor beta (TGFβ) (Schonborn et al., 2020; Sun et al., 2022).

Like collagen VI, collagen XII is localized pericellularly in many tissues (Izu et al., 2011; Izu et al., 2021). In this capacity, collagen XII is thought to form collagen bridges that may facilitate intercellular communication, cell orientation and storage of bioactive ligands (Izu and Birk, 2023), a notion supported by the observation that osteoblast polarity and maturation is severely compromised in collagen XII - deficient mice (Izu et al., 2011). Studies in zebrafish suggest intercellular collagen XII bridging may also play a key role in the promotion of axon extension across experimentally-induced spinal cord injuries (Wehner et al., 2017) and facilitating the migration of cardiomyocytes into injured tissues during regeneration of cryo-damaged heart (Marro et al., 2016). Interestingly, colocalization of collagen XII and VI has been reported in the pericellular zones of osteoblast cultures (Izu et al., 2016) and in regenerative axons (Wehner et al., 2017) suggesting a functional relationship between the two collagens. Indeed, co-regulation of collagen VI and XII gene expression has been reported in human mesenchymal stem cells (MSCs) (McNeill et al., 2020) and in zebrafish (Wehner et al., 2017) suggesting the potential need for stoichiometric regulation between the two collagens.

#### 1.3.4 Collagen VI mutations and disease

Dominant and recessive mutations in collagen XII cause an Ehler’s Danlos Syndrome (EDS) - like pathology in humans characterized primarily by joint hypermobility, contractures, and abnormal skin healing (Izu and Birk, 2023). These symptoms are frequently overlapped by features resembling Ullrich congenital muscular dystrophy, taking the form of a gradually progressive muscle disease characterized by muscle contractures, motor dysfunction, and weakness (Hicks et al., 2014). This complex disease phenotype has since been referred to as myopathic-type EDS (mEDS) (Malfait et al., 2017; Malek and Koster, 2021), associated with a range of mEDS mutations that perturb collagen XII translation, processing or secretion resulting in weakened and dysfunctional ECM that accounting for a disease phenotype of varying severity (Araujo and Antunes, 2021; Malek and Koster, 2021; Coppens et al., 2022; Furuhata-Yoshimura et al., 2023; Izu and Birk, 2023; Zhu et al., 2023). The mEDS phenotype has been closely recapitulated in collagen XII–deficient mice (Zhu et al., 2023).

## 2 Hypothesis and discussion: genetic and biochemical evidence supports a functional physical interaction between collagen VI and collagen XII

**FIGURE 3:**
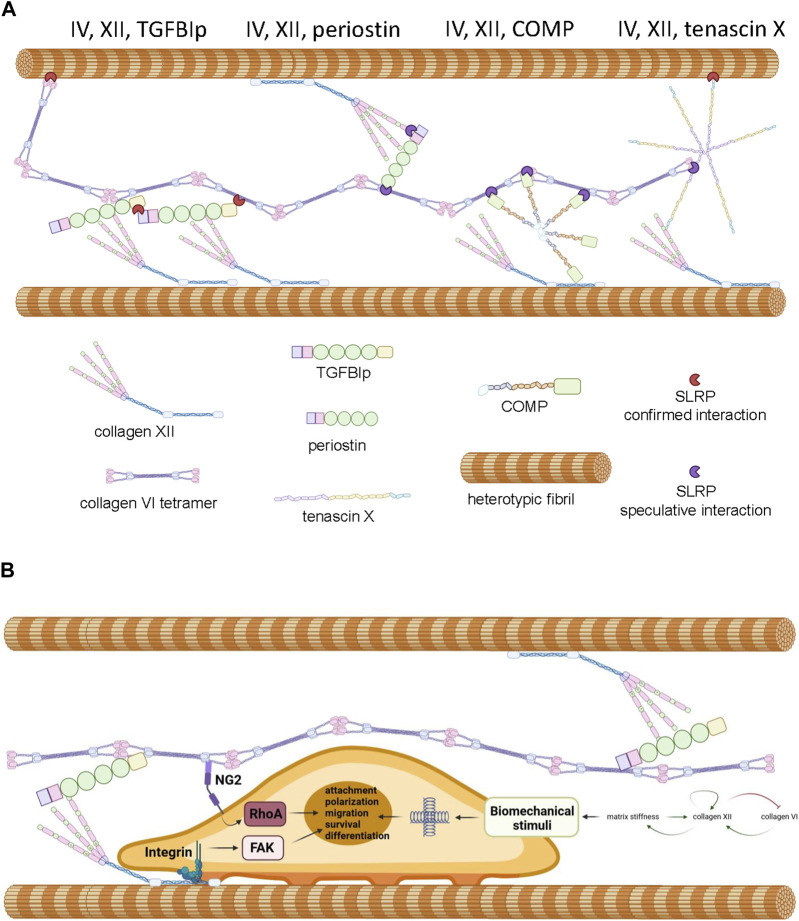
**Panel (A)** Proposed model of interactions between collagen VI, collagen XII, heterotypic fibrils and accessory molecules as described in the text. Note that SLRPs presented in red represent experimentally confirmed interactions and SLRPs presented in purple represent speculated interactions predicted by the literature. **Panel (B)** Proposed mechanochemical consequences of interactions between collagen VI, collagen XII, TGFIp and attached cells. Interaction with the complex is predicted to trigger FAK and RhoA signaling contributing to cell polarization, survival, and tissue-specific processes. Crosslinking between heterotypic fibrils results in increased stiffness which triggers mechanosensory responses including the modulation of collagen XII and collagen VI expression. Generated by Biorender, representations of structure are simplified and molecules are not drawn to scale.

The overlapping phenotypes in the mixed myopathy/Ehlers-Danlos syndrome spectrum of diseases suggests a close functional relationship between collagen VI and XII (Delbaere et al., 2020). Indeed, immunohistochemical colocalization has been demonstrated in cultured osteoblasts (Izu et al., 2016), MSCs (McNeill et al., 2020), and connective tissues of developing zebrafish (Tonelotto et al., 2019). While circumstantial, these data do suggest the possibility of an ECM complex where direct interaction, or indirect interaction via accessory proteins occurs between collagen VI and XII, facilitating coordinated physiological activities. In support of this hypothetical functional network (Figure 3A), it is known that collagen XII interacts with heterotypic fibrils via its carboxyl-terminal NC1 and collagenous domains (Figure 2C) while simultaneously forming tight associations with tenascin X, a 450 kDa multi-domain glycoprotein (Miller, 2020), via the amino-terminal NC3 domain (Veit et al., 2006; Delbaere et al., 2020). Tenascin X also has the capacity to interact with heterotypic fibrils through small leucine rich accessory proteins (SLRPs) such as decorin (Font et al., 1996; Schaefer and Iozzo, 2008; Delbaere et al., 2020). On the other hand, collagen VI has the capacity to interact with heterotypic fibrils via the protruding globular domains of collagens (Bonod-Bidaud et al., 2012) and decorin (Nareyeck et al., 2004). These confirmed interactions offer theoretical support for the existence an indirect collagen VI/XII complex, but direct interaction between collagen VI and XII has not been demonstrated and a single accessory protein that could physically bridge them has not yet been identified. Nevertheless, there are candidates for bridging molecules that might have the potential to serve as an interface between collagen XII and VI, and coordinate their functionality.

### 2.1 Transforming growth factor beta induced protein

Transforming growth factor beta induced protein ig-h3, also referred to as TGFBIp, β-ig-H3, and keratoepithelin is induced by TGFβ and known to participate in morphogenesis, angiogenesis, cell attachment, tissue healing and inflammation (Thapa et al., 2007). The *TGFBI* gene encodes a 78 kDa protein (TGFBIp) consisting of four fascilin-1 (FAS1) domains and four integrin binding domains but this protein is cleaved upon secretion to 68 kDa (Thapa et al., 2007; Ween et al., 2012). *TGFBI* mutations have not been associated with the mEDS spectrum of diseases but they are prevalent in corneal dystrophy, affecting the biomechanical and optical properties of the tissue (Thapa et al., 2007). TGFBIp is also a linker protein, with the capacity to bind via disulphide bridging and non-covalently to collagen VI fibrils (Hanssen et al., 2003) and also via ternary complexes with SLRPs (Reinboth et al., 2006). TGFBIp also has the capacity to covalently bind to collagen XII via disulphide bridging (Runager et al., 2013). While the binding sites of collagen XII and VI on TGFBIp have not been delineated, the tendency of TGFBIp to form higher order complexes with itself and SLRPs suggests it could simultaneously interact with collagen XII and VI even if the binding sites overlap (Hanssen et al., 2003; Reinboth et al., 2006). The physiological function of a putative complex between pTGFBI, collagen VI and collagen XII is unclear, but it is theoretically possible that the complex serves to ensure attached cells receive coordinated stimuli and that these signals are compatible with correct cellular orientation (Figure 3B). Indeed, a role for collagen VI, XII and TGFBI complexes in maintenance of cellular orientation is supported by the observation that collagen XII and collagen VI are both essential for maintaining osteoblast polarity and proper alignment on the trabecular surface (Izu et al., 2011; Izu et al., 2012), TGFBIp deficiency results in aberrant bone growth (Lee et al., 2015), and osteoinductive ECM preparations generated from MSCs rely on the presence of type VI and type XII collagen, and frequently possess high levels of TGFBIp (McNeill et al., 2020). The biochemical stimuli delivered by complexed collagen VI, XII, and TGFBIp to cells are likely to be extensive, but when bound to collagen XII, TGFBIp activates the focal adhesion kinase (FAK) axis via engagement of integrin receptors (Grigoriou et al., 2005; Runager et al., 2013). Furthermore, collagen VI fibrils signal to cells via nerve/glial antigen 2 (NG2) (Russell et al., 2013) to activate the Rho axis (Biname, 2014) and Akt-mediated survival pathways (Iyengar et al., 2005). It is noteworthy that coordinated activation of NG2 and FAK signaling is known to enhance cell attachment, migration and cell polarity in several systems (Ridley, 2000; Biname, 2014; Sardone et al., 2016). Given the role collagen XII plays in the regulation of ECM biomechanics and the role collagen VI plays in the transduction of polarity and migration signals, it attractive to hypothesize that complexes of collagen VI, collagen XII, and TGFBIp exist to fine tune the migratory behavior of cells in response to the biomechanical environment within the pericellular space and beyond (Figure 3B). In consideration of this hypothesis, it is important to note that dysregulated collagen XII and VI expression predicts aberrant migratory behavior of tumor cells and poor prognosis in colorectal cancers (Wu and Xu, 2020), ECM rich in collagen XII, VI, and TGFBIp stimulates osteoprogenitor retention and osteogenic repair (Zeitouni et al., 2012) and collagen VI and XII simultaneously play a role in the facilitation of cardiomyocyte migration (Marro et al., 2016) and axonal outgrowth (Wehner et al., 2017) during tissue regeneration.

### 2.2 Periostin

Periostin is a paralog of TGFBIp (Thapa et al., 2007), consisting of 4 FAS1 domains and an emilin-like EMI domain that facilitates binding to collagen I, collagen V, fibronectin, and notch-1 (Dorafshan et al., 2022). Periostin’s homology to TGFBIp suggests that it could also serve as a bridging molecule with collagen VI and XII. While direct interaction between collagen VI, collagen XII and periostin has not yet been demonstrated, interaction of both collagens with periostin via SLRPs is theoretically possible (Figure 3A). TGFBIp and periostin have the capability to interact with one another via EMI domains and heteromultimers are secreted by COS-7 cells (Kim et al., 2009). While bound to periostin, TGFBIp retains its ability to bind collagen VI but not *vice versa* (Kim et al., 2009). TGFBIp and periostin is always present in osteogenic cell matrices generated by MSCs (McNeill et al., 2020), suggesting that heteromultimerization might be necessary to ensure proper secretion and assembly into higher order structures. Future knock-down experiments in MSCs and osteogenic assays on the resultant matrices could shed light on this question. It should be noted that periostin does not have the capacity to complement against corneal dysfunction in TGFBIp-deficient mice (Poulsen et al., 2018).

### 2.3 Tenascin

The tenascins are a 4-member family of oligomeric proteins (termed C,X, R and W) that share a high degree of structural homology but distinctive tissue distribution (Hsia and Schwarzbauer, 2005). Tenascins are modular proteins consisting of amino-terminal heptad repeats, epidermal growth factor (EGF)-like repeats, F3 repeats and a carboxyl-terminal fibrinogen-like domain with several inter-dispersed integrin binding sites (Hsia and Schwarzbauer, 2005). Exposure of cultured cells to tenascins cause the formation of filapodia and regulate cell motility (Wenk et al., 2000). Tenascin X is known to bind to the collagen XII NC3 domain and also directly to heterotypic fibrils via SLRPs (Font et al., 1996; Veit et al., 2006). *In vitro* solid-phase assays, collagen VI failed to interact with tenascin X (Minamitani et al., 2004a), but bridging by tenascin X between collagen XII and collagen VI is theoretically possible via ternary interactions with SLRPs (Font et al., 1996; Elefteriou et al., 2001; Nareyeck et al., 2004; Reinboth et al., 2006; Delbaere et al., 2020) (Figure 3A). Expression of tenascin X and collagen VI also appears to be coordinately regulated (Minamitani et al., 2004b) and they collaborate in driving the formation of heterotypic fibrils containing collagen I (Minamitani et al., 2004a). Tenascin X deficiency also causes a spectrum of mEDS-like disorders that mimic the phenotype of collagen VI related myopathies (Burch et al., 1997; Brisset et al., 2020; Marino et al., 2022; Okuda-Ashitaka and Matsumoto, 2023).

### 2.4 Cartilage oligomeric matrix protein

Cartilage oligomeric matrix protein (COMP) also known as thrombospondin-5, is a member of the thrombospondin family of multi-domain ECM proteins primarily expressed in connective and skeletal tissues. COMP facilitates the secretion and proper assembly of collagen into fibrils (Schulz et al., 2016), and serves to stabilize the ECM through a range of interactions with collagens, membrane proteins, proteoglycans, and ligands of the TGFβ superfamily (Cui and Zhang, 2022). COMP has been shown to directly interact with collagen XII (Agarwal et al. 2012), but there is currently no evidence to suggest it has the capacity to interact directly with collagen VI (Acharya et al., 2014). Interaction between COMP and type VI collagen is however possible via SLRPs and matrillin (Mann et al., 2004).

## 3 Summary and outlook

It is clear that collagens VI and XII possess intrinsic properties that promote cell survival, proper cellular polarity, structural support of tissue architecture and cellular differentiation and tissue maintenance. Furthermore, the substantial body of genetic data in humans and from cell and biochemical studies, indicates a strong functional overlap between the two collagens and the feasibility of synergistic functionality via direct or indirect physical interactions.

The reason for a lack of data on the existence of interactions between collagen XII and VI is unclear given the overlapping phenotypes of mutations in humans and in transgenic mice, but biochemical assays are likely to be complex because the collagens are challenging to purify from tissues and difficult to generate recombinantly in useful quantities. Nevertheless, protocols do exist that describe isolation of wild type collagen VI and XII (Takasaki et al., 1995; Beecher et al., 2011), and these molecules can be visualized by electron microscopy (Watt et al., 1992; Koch et al., 1995) With pure isolates of type VI and type XII collagen, it is theoretically possible to perform surface plasmon resonance and simple solid-phase binding assays. However, it is currently unclear whether type XII collagen must be incorporated into a fibril, or type VI collagen must generate beaded microfibrils, or whether ancillary proteins need to be included to initiate ternary complexes, and with greater complexity, characterization of direct or ternary interaction becomes more challenging. If direct molecular interaction between collagen XII and VI cannot be demonstrated using biochemical approaches, it is likely that high-power EM or cryo-EM will be necessary to visualize complexes. Another approach might be to perform binding assays with recombinantly expressed domains derived from the collagens. Given that colα1−6(VI) possesses 4–16 domains, and colα1 (XII) possesses 27 domains, there are 432 possible permutations and the potential for multimerization or higher-order structure formation is not recapitulated in these simple systems. It should also be noted that the clinical collagen XII mutations cause cell-retention and/or collagen XII deficiency (OMIM 120320), offering no useful information on potential sites for molecular interactions. While experiments utilizing full-length and truncated molecules have their limitations, it seems that a combination of these approaches is warranted.

This article summarizes the evidence for the existence of biological interactions between collagen VI and XII whether directly or indirectly through candidate bridging molecules such as TGFBIp, periostin and tenascin X. Given their overlapping roles in the homeostatic regulation and regeneration of a broad range of tissues, a comprehensive understanding of the apparent mechanistic relationship between collagen VI and XII has the potential to contribute significantly to our understanding of tissue homeostasis, wound healing and disease pathology. One broader impact of this avenue of investigation is the potential for generation of therapeutic ECM products with the capacity to heal a range of tissues.

## Figures and Tables

**Table:** 

**Myopathic Ehler’s Danlos Syndrome**	A rare systemic disease characterized by congenital muscle hypotonia and/or muscle atrophy that improves with age, proximal joint contractures (knee, hip, elbow), and hypermobility of distal joints
**Glycoside**	A molecule in which a sugar is bound to another functional group via a glycosidic bond
**Fibronectin Type III**	Fibronectin is a dimeric glycoprotein composed of disulfide-linked subunits. They are involved in cell adhesion, cell morphology, thrombosis, cell migration, and embryonic differentiation. Type III repeats are both the largest and the most common of the fibronectin subdomains
**Kunitz domains**	Kunitz domains are the active domains of proteins that inhibit the function of protein degrading enzymes or, more specifically, domains of Kunitz-type are protease inhibitors
**Thrombospondin-1**	Thrombospondin-1 is a secreted matricellular glycoprotein that modulates cell behavior by interacting with components of the extracellular matrix and with several cell surface receptors
**von Willebrand factor A (VWA)**	The von Willebrand A (VWA) domain is iinvolved in cell adhesion, extracellular matrix proteins, and in integrin receptors.
**Endotrophin**	Endotrophin is a cleavage product derived from the collagen VI(α3) chain
**Chondrogenesis**	Chondrogenesis is the biological process through which cartilage tissue is formed and developed
**Chondroitin Sulphate Proteoglycan 4 (CSPG4**	Chondroitin sulfate proteoglycan 4 (CSPG4) is a multifunctional transmembrane proteoglycan involved in spreading, migration and invasion of melanoma
**Platelet derived growth factor (PDGF)**	Platelet derived growth factor (PDGF) constitutes a family of dimeric isoforms, acting on connective tissue cells and certain other cell types
**OncostatinM (OSM)**	OSM is a pleiotropic cytokine that belongs to the interleukin 6 group of cytokines
**Bethlem muscular dystrophy**	Bethlem myopathy is a form of muscular dystrophy that causes joint stiffness and muscle weakness that gradually becomes worse over time. It often affects the feet, hands and elbows
**Ullrich congenital muscular dystrophy (UCMD)**	UCMD is a form of congenital muscular dystrophy with specific features. There are three genes responsible for UCMD: they are called COL6A1, COL6A2 and COL6A3, and they carry the genetic blueprint that is used to produce a protein called collagen VI.
**Retinitis Pigmentosa (RP)**	Retinitis pigmentosa (RP) is a group of rare eye diseases that affect the retina (the light-sensitive layer of tissue in the back of the eye). RP makes cells in the retina break down slowly over time, causing vision loss
**Atopic Dermatitis**	Atopic dermatitis, often referred to as eczema, is a chronic (long-lasting) disease that causes inflammation, redness, and irritation of the skin
**Tenascin-X (TNX)**	Tenascin-X is the largest member of the tenascin (TN) family of evolutionary conserved extracellular matrix glycoproteins, which also comprises TN-C, TN-R and TN-W. Among this family, TN-X is the only member described so far to exert a crucial architectural function as evidenced by a connective tissue disorder (a recessive form of Ehlers-Danlos syndrome) resulting from a loss-of-function of this glycoprotein in humans and mice.
**Decorin**	Decorin is a stromal proteoglycan synthesized chiefly by fibroblasts, stressed vascular endothelial cells, and smooth muscle cells. A member of the SLRP family
**Fasciclin-1 (FAS1)**	The Fasciclin 1 (FAS1) domain is an ancient structural motif in extracellular proteins present in all kingdoms of life and particularly abundant in plants. The FAS1 domain accommodates multiple interaction surfaces, enabling it to bind different ligands
**Focal adhesion kinase (FAK)**	Focal adhesion kinase (FAK) is a crucial signalling component that is activated by numerous stimuli and functions as a biosensor or integrator to control cell motility
**Periostin**	Periostin is an osteoblast-specific factor that is expressed in collagen-rich fibrous connective tissues such as bone, heart valves, tendons, and periodontal ligaments, as well as by some tumors
**Filopodia**	Filopodia are thin, actin-rich plasma-membrane protrusions that function as antennae for cells to probe their environment
**Isolated recessive dystonia**	Early-onset isolated dystonia is characterized by involuntary muscle contractions, twisting of specific body parts such as an arm or a leg, tremors, and other uncontrolled movements
**Myoclonus epilepsy syndrome**	Myoclonic seizures are characterized by rapid, jerk like movements that can affect the face, limbs, or axial musculature
**Fibrillar Collagens**	Fibrillar collagens functions are to provide three-dimensional frameworks for tissues and organs. These networks confer mechanical strength as well as signalling and organizing functions through binding to cellular receptors and other components of the ECM.
**Small leucine rich accessory proteins (SLRP)**	Proteoglycans consisting of a protein core with leucine rich motifs covalently linked to glycosaminoglycan side chains. Usually found associated with ECM components
**Heterotypic fibril**	Fibrils consisting of mixtures of collagen trimers laterally arranged and crosslinked to form rope-like structures with high tensile strength. They are composed of one or two quantitatively major collagens (I–III) as well as one minor collagen (V or XI)
**Matrisome**	A complete list of proteins in a given extracellular matrix

## Data Availability

The original contributions presented in the study are included in the article/Supplementary Material, further inquiries can be directed to the corresponding author.
