# Supplementary material for: The coordinated activities of collagen VI and XII in maintenance of tissue structure, function and repair: evidence for a physical interaction
Source: Front Mol Biosci. 2024 Mar 28;11:1376091. doi: 10.3389/fmolb.2024.1376091 (PMC11007232; doi:10.3389/fmolb.2024.1376091)
Supplement: Supplementary file 1 [file Table1.DOCX]

**Glossary of Terms.**

| **Myopathic Ehler’s Danlos Syndrome** | A rare systemic disease characterized by congenital muscle hypotonia and/or muscle atrophy that improves with age, proximal joint contractures (knee, hip, elbow), and hypermobility of distal joints. |
| --- | --- |
| **Glycoside** | A molecule in which a sugar is bound to another functional group via a glycosidic bond |
| **Fibronectin Type III** | Fibronectin is a dimeric glycoprotein composed of disulfide-linked subunits. They are involved in cell adhesion, cell morphology, thrombosis, cell migration, and embryonic differentiation. Type III repeats are both the largest and the most common of the fibronectin subdomains. |
| **Kunitz domains** | Kunitz domains are the active domains of proteins that inhibit the function of protein degrading enzymes or, more specifically, domains of Kunitz-type are protease inhibitors. |
| **Thrombospondin-1** | Thrombospondin-1 is a secreted matricellular glycoprotein that modulates cell behavior by interacting with components of the extracellular matrix and with several cell surface receptors. |
| **von Willebrand factor A (VWA)** | The von Willebrand A  (VWA) domain is iinvolved in cell adhesion, extracellular matrix proteins, and in integrin receptors. |
| **Endotrophin** | Endotrophin is a cleavage product derived from the collagen VI(α3) chain. |
| **Chondrogenesis** | Chondrogenesis is the biological process through which cartilage tissue is formed and developed. |
| **Chondroitin Sulphate Proteoglycan 4 (CSPG4** | Chondroitin sulfate proteoglycan 4 (CSPG4) is a multifunctional transmembrane proteoglycan involved in spreading, migration and invasion of melanoma. |
| **Platelet derived growth factor (PDGF)** | Platelet-derived growth factor (PDGF) constitutes a family of dimeric isoforms, acting on connective tissue cells and certain other cell types. |
| **OncostatinM (OSM)** | OSM is a pleiotropic cytokine that belongs to the interleukin 6 group of cytokines. |
| **Bethlem muscular dystrophy** | Bethlem myopathy is a form of muscular dystrophy that causes joint stiffness and muscle weakness that gradually becomes worse over time. It often affects the feet, hands and elbows. |
| **Ullrich congenital muscular dystrophy (UCMD)** | UCMD is a form of congenital muscular dystrophy with specific features. There are three genes responsible for UCMD: they are called COL6A1, COL6A2 and COL6A3, and they carry the genetic blueprint that is used to produce a protein called collagen VI. |
| **Retinitis Pigmentosa (RP)** | Retinitis pigmentosa (RP) is a group of rare eye diseases that affect the retina (the light-sensitive layer of tissue in the back of the eye). RP makes cells in the retina break down slowly over time, causing vision loss. |
| **Atopic Dermatitis** | Atopic dermatitis, often referred to as eczema, is a chronic (long-lasting) disease that causes inflammation, redness, and irritation of the skin. |
| **Tenascin-X (TNX)** | Tenascin-X is the largest member of the tenascin (TN) family of evolutionary conserved extracellular matrix glycoproteins, which also comprises TN-C, TN-R and TN-W. Among this family, TN-X is the only member described so far to exert a crucial architectural function as evidenced by a connective tissue disorder (a recessive form of  Ehlers-Danlos syndrome) resulting from a  loss-of-function of this glycoprotein in humans and mice. |
| **Decorin** | Decorin is a stromal proteoglycan synthesized chiefly by fibroblasts, stressed vascular endothelial cells, and smooth muscle cells. A member of the SLRP family. |
| **Fasciclin-1 (FAS1)** | The Fasciclin 1 (FAS1) domain is an ancient structural motif in extracellular proteins present in all kingdoms of life and particularly abundant in plants. The FAS1 domain accommodates multiple interaction surfaces, enabling it to bind different ligands. |
| **Focal adhesion kinase (FAK)** | Focal adhesion kinase (FAK) is a crucial signalling component that is activated by numerous stimuli and functions as a biosensor or integrator to control cell motility. |
| **Periostin** | Periostin is an osteoblast-specific factor that is expressed in collagen-rich fibrous connective tissues such as bone, heart valves, tendons, and periodontal ligaments, as well as by some tumors. |
| **Filopodia** | Filopodia are thin, actin-rich plasma-membrane protrusions that function as antennae for cells to probe their environment. |
| **Isolated recessive dystonia** | Early-onset isolated dystonia is characterized by involuntary muscle contractions, twisting of specific body parts such as an arm or a leg, tremors, and other uncontrolled movements. |
| **Myoclonus epilepsy syndrome** | Myoclonic seizures are characterized by rapid, jerk like movements that can affect the face, limbs, or axial musculature. |
| **Fibrillar Collagens** | Fibrillar collagens functions are to provide  three-dimensional frameworks for tissues and organs. These networks confer mechanical strength as well as signalling and organizing functions through binding to cellular receptors and other components of the ECM. |
| **Small leucine rich accessory proteins (SLRP)** | Proteoglycans consisting of a protein core with leucine rich motifs covalently linked to glycosaminoglycan side chains. Usually found associated with ECM components. |
| **Heterotypic fibril** | Fibrils consisting of mixtures of collagen trimers laterally arranged and crosslinked to form rope-like structures with high tensile strength. They are composed of one or two quantitatively major collagens (I–III) as well as one minor collagen (V or XI). |
| **Matrisome** | A complete list of proteins in a given extracellular matrix. |
